# Axially Chiral Organic Semiconductors for Visible‐Blind UV‐Selective Circularly Polarized Light Detection

**DOI:** 10.1002/advs.202308262

**Published:** 2024-02-04

**Authors:** Yejin Kwon, Je‐Yeon Jung, Won Bo Lee, Joon Hak Oh

**Affiliations:** ^1^ School of Chemical and Biological Engineering Institute of Chemical Processes Seoul National University 1 Gwanak‐ro, Gwanak‐gu Seoul 08826 Republic of Korea

**Keywords:** binaphthyl, chirality, circularly polarized light, organic phototransistors, self‐assembly

## Abstract

Technologies that detect circularly polarized light (CPL), particularly in the UV region, have significant potential for various applications, including bioimaging and optical communication. However, a major challenge in directly sensing CPL arises from the conflicting requirements of planar structures for efficient charge transport and distorted structures for effective interaction with CPL. Here, a novel design of an axially chiral *n*‐type organic semiconductor is presented to surmount the challenge, in which a binaphthyl group results in a high dissymmetry factor at the molecular level, while maintaining excellent electron‐transporting characteristics through the naphthalene diimide group. Experimental and computational methods reveal different stacking behaviors in homochiral and heterochiral assemblies, yielding different structures: Nanowires and nanoparticles, respectively. Especially, the homochiral assemblies exhibit effective π–π stacking between naphthalene diimides despite axial chirality. Thus, phototransistors fabricated using enantiomers exhibit a high maximum electron mobility of 0.22 cm^2^ V^−1^ s^−1^ and a detectivity of 3.9 × 10^12^ Jones, alongside the CPL distinguishing ability with a dissymmetry factor of responsivity of 0.05. Furthermore, the material possesses a wide bandgap, contributing to its excellent visible‐blind UV‐selective detection. These findings highlight the new strategy for compact CPL detectors, coupled with the demonstration of less‐explored *n*‐type and UV region phototransistors.

## Graphical abstract

An organic semiconductor with axial chirality is proposed by linking two naphthalene diimides with a binaphthyl group. This innovative material demonstrates distinctive chiral self‐discrimination behavior and assembles into nanowires. It has potential applications as a phototransistor for selectively detecting circularly polarized light (CPL) in the UV region, in conjunction with possessing high electron mobility and excellent detectivity.

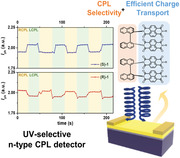

## 1 Introduction

CPL plays a pivotal role in various advanced applications^[^ ^1^ ^]^ including optical quantum computing,^[^ ^2^ ^]^ encrypted communications,^[^ ^3^ ^]^ and bioimaging.^[^ ^4^ ^]^ In these domains, the need for CPL detectors that can selectively distinguish between the two polarization states of CPL is crucial. A conventional method to detect CPL involves the use of silicon photodetectors combined with additional optical apparatus, that is, a linear polarizer and a phase retarder. However, there is a growing necessity for materials capable of directly and inherently sensing CPL without requiring additional apparatus. Such materials are essential for advancing the development of miniaturized and integrated CPL detectors. Researchers have explored various CPL‐responsive materials,^[^ ^5^ ^]^ encompassing inorganic nanomaterials,^[^ ^6^ ^]^ chiral organic small molecules, polymers,^[^ ^7^ ^]^ and hybrid organic‐inorganic perovskites.^[^ ^8^ ^]^ Among them, organic semiconductors exhibit distinct advantages over other materials due to their intrinsic chirality, lightweight, cost‐effectiveness, and the ease with which their chiroptical properties can be tailored through rational molecular design.

To date, several approaches have been reported to fabricate organic CPL detectors, such as blending achiral polymers with chiral small molecular additives,^[^ ^9^ ^]^ amplifying chirality by supramolecular or multiscale assemblies,^[^ ^10^ ^]^ and utilizing twisted molecules.^[^ ^11^ ^]^ However, there is an inevitable trade‐off between charge transport properties and CPL selectivity, as the former requires planar π‐stacked structures while the latter requires distorted structures.^[^ ^12^ ^]^ Therefore, most previously reported organic CPL detectors show low optoelectronic properties and limited CPL selectivity. Thus, there is a demand for the development of chiral organic semiconductors that can sense CPL selectively while maintaining efficient charge transport.

In the development of CPL detectors, the targeted wavelength is also an important factor to be taken into consideration. Notably, UV light holds immense promise across various fields. For instance, the selective detection of CPL in the UV range proves invaluable in biological diagnostics. Certain biological components display circular polarization responses only under UV light, enabling precise qualitative and quantitative analysis.^[^ ^13^ ^]^ Interestingly, some animals, like the mantis shrimp, possess the ability to discern the circular polarization state of light, leveraging the UV spectrum for perception and communication.^[^ ^14^ ^]^ Similarly, a CPL detector operating in the UV range could pave the way for expanding beyond human vision, potentially unlocking novel applications in optical communication.

Despite its potential, the majority of previous research on organic CPL detectors has focused on the visible or near‐infrared light spectrum, with only a limited number of studies addressing the UV region. For example, Campbell et al.^[^ ^11a^ ^]^ reported a CPL detector utilizing 1‐aza[6]helicene, demonstrating selective detection of 365 nm light. However, for practical applications, further enhancements in electronic properties are necessary, given the maximum hole‐transport mobility of 10^−4^ cm^2^ V^−1^ s^−1^ and an on/off ratio of 10^3^. More recently, Lim et al.^[^ ^9d^ ^]^ and Qin et al.^[^ ^9e^ ^]^ independently reported organic chiroptical detectors capable of distinguishing UV CPL with improved performance. Nevertheless, these devices respond across a broad spectral range of CPL and thus lack selectivity for UV light. Hence, it is crucial to develop CPL detectors that possess i) high circular polarization selectivity, ii) efficient charge transport, and iii) exclusive responsiveness within the UV range, without interference from room lighting.

In this context, we designed a novel strategy to achieve these characteristics: linking two organic semiconductors, naphthalene diimides (NDIs), using a 1,1′‐binaphthyl (BN) group. BN is an atropisomer with a large chiral pitch that matches the specific handedness of CPL. Due to the steric repulsion of the planar transition state, their enantiomers have a large rotational barrier that cannot be easily interconverted. BN derivatives have been widely studied in the fields of asymmetric catalysis^[^ ^15^ ^]^ and chiral self‐assembly.^[^ ^16^ ^]^ However, their importance in the field of organic electronics has been relatively overlooked, despite their potential for interacting with CPL as a chiral active layer.^[^ ^17^ ^]^ To enhance charge transport, we linked two NDIs to BN, as NDIs possess promising characteristics as *n*‐type organic optoelectronic materials, such as high electron affinity, strong π‐planar stacking, high charge carrier mobility, and excellent thermal and chemical stability.^[^ ^18^ ^]^

**Scheme 1:**
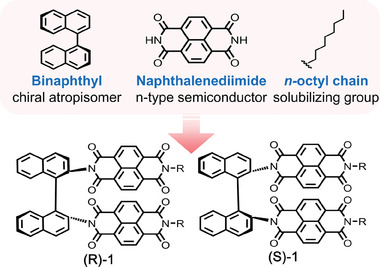
Schematic illustration of molecular design strategy of (R)−1 and (S)−1. BN was intended to serve as a chiral scaffold, NDI as an *n*‐type semiconductor, and the *n*‐octyl chain as a solubilizing group.

With the aforementioned rationale, here, we report a novel axially chiral *n‐*type organic semiconductor (i.e., (R)−1 and (S)−1 in **Scheme** **1**) and its applications to organic phototransistors (OPTs) for UV CPL sensing. Upon self‐assembly, the enantiomers, (R)−1 and (S)−1, formed 1D nanowires (NWs), whereas the racemates formed nanoparticles (NPs). The enantiomer exhibited structurally favored molecular stacking with a shorter π–π distance and a longer assembly length compared to the racemate, as molecules of the same chirality come together to form a well‐defined 1D structure.^[^ ^19^ ^]^ Intriguingly, self‐assembly at different enantiomeric ratios, coupled with thermodynamic analysis using computational methods, revealed a preference for chiral self‐discrimination.

We also investigated the optoelectrical properties of (R)−1 and (S)−1 in thin film states. Interestingly, the fabricated OPTs exhibited high electron mobility of up to 0.22 cm^2^ V^−1^ s^−1^, despite the highly distorted molecular structure. Through this molecular design, we achieved a wide bandgap, enabling UV‐selective sensing with a maximum detectivity of 3.9 × 10^12^ Jones under illumination with light at a wavelength of 385 nm. The OPTs clearly discriminated the handedness of the CPL with a dissymmetry factor of responsivity (*g*_R_) of 0.05, indicating their high potential for use in UV CPL detectors. We anticipate that the synergistic combination of the excellent optoelectronic properties of NDIs and the rich BN chemistry will open a new avenue for the development of a new class of organic CPL detectors.

## 2 Results and Discussion

### 2.1 Synthesis and Self‐Assembly

We, for the first time, synthesized BN‐linked NDIs, that is, (R)−1 and (S)−1 (Scheme 1). The molecular structures were rationally designed, that is, BN played a role as a chiral scaffold, NDI as an *n*‐type semiconductor, and the *n*‐octyl chain as a solubilizing group for solution processing. They were synthesized by sequentially reacting naphthalene‐1,4,5,8‐tetracarboxylic dianhydride with *n*‐octyl amine and (R)‐ or (S)−1,1′‐binaphthyl‐2,2′‐diamine (Scheme S1, Supporting Information). Detailed synthetic procedures and characterization are described in the Supporting Information.

**Figure 1:**
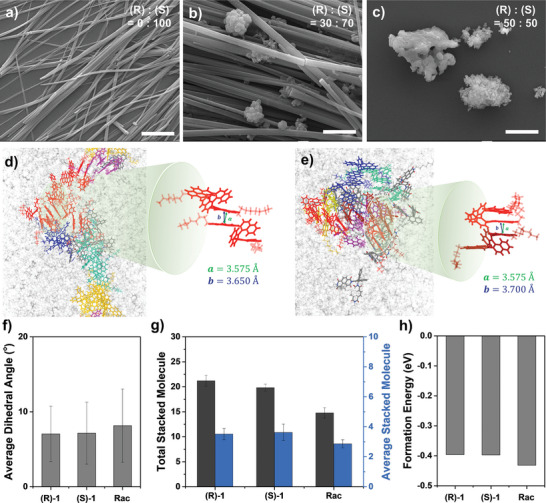
Chiral self‐assembly behavior. SEM images of self‐assembled structures in enantiomeric excess of a) 100%, b) 40%, and c) 0% (scale bars: 3 µm). Snapshots from MD simulation indicating the finalized configuration of the self‐assembly of d) (S)−1 and e) racemate. Stacked clusters are depicted in the same color, while unstacked structures are indicated in opaque gray and MCH is indicated in transparent gray. f) Average dihedral angles of (R)−1, (S)−1, and racemate. g) Numbers of total (left) and average (right) stacked molecules in (R)−1, (S)−1, and racemate. Note that the stacking criterion was 5.00 Å between the COG of the NDIs. h) Formation energy of (R)−1, (S)−1, and racemate upon stacking.

To study the chiral self‐sorting behavior, self‐assembled structures of (R)−1 and (S)−1 were grown using a nonsolvent nucleation method. (R)−1 and (S)−1 were soluble in most organic solvents, while they remained partially soluble in methylcyclohexane (MCH). They were dissolved in MCH (1 mg mL^−1^) at 90 °C, and subsequent cooling to room temperature prompted the recrystallization of molecules due to the decreased solubility. Similar to previous reports with chiral rylene diimides, the enantiomer and racemate showed different nanostructures.^[^ ^16^, ^19^, ^20^ ^]^ The pure enantiomer (**Figure** **1**a) formed crystalline 1D NWs with a diameter of 862 (± 158) nm and lengths of the order of hundreds of micrometers. In contrast, the racemate (Figure 1c) formed ill‐defined NPs. Transmission electron microscope (TEM) images and selected area electron diffraction (SAED) patterns illustrated that NWs derived from a pure enantiomer had a highly crystalline structure, in contrast to the amorphous nature of NPs formed from the racemate (Figure S1, Supporting Information). The *d*‐spacing values of NWs were determined to be 3.64 Å, which aligned with the characteristic π–π spacing. However, discerning the precise molecular arrangement of crystal structures from SAED data posed a challenge due to the strong impact of the electron beam on the NWs, resulting in damage. Then, we analyzed the coassembly of (R)−1 and (S)−1 with varying enantiomeric excess (ee). In optical microscopy (OM) and scanning electron microscopy (SEM) images of the coassembly of (R)−1 and (S)−1 with different ratios, both NPs and NWs were observed (Figure 1b and Figure S3, Supporting Information). The percentage of NWs increased as the ee increased from 0% to 100%, while the percentage of NPs decreased. Therefore, we inferred that NWs were formed in homochiral assembly and NPs were formed in heterochiral assembly. These observations indicated that self‐discrimination prevailed over self‐recognition for the racemates. Furthermore, as the assembly gradually shifted from NPs to NWs, the dissymmetry factor of absorption (*g*_abs_) value also increased (Figure S4, Supporting Information). The *g*_abs_ values were measured by circular dichroism (CD) spectroscopy using the following equation,

(1) $$g_{abs}=\frac{\Delta A}{A}=\frac{\Theta }{33000\times A}$$

where Δ*A* and *A* represent the differential and average absorbance of left‐ and right‐handed CPL, respectively, and Θ indicates the ellipticity. The maximum *g*_abs_ values were −2.2  ×  10^−3^ and 2.1  ×  10^−3^ for enantiomeric (R)−1 and (S)−1.

### 2.2 Computational Analysis

Molecular dynamics (MD) simulations were conducted to elucidate the difference in self‐assembly processes between enantiomers and racemates in MCH. After running a 170 ns NVT simulation, the last 20 ns of data were used to analyze stacking structures. The assembly of (R)−1 and (S)−1 constructed a more well‐stacked structure than an assembly of the racemate, which could be interpreted by the height of the first peak of the radial distribution functions (RDFs) with center of geometry (COG) distance between NDIs (Figure S5, Supporting Information). The first π–π interaction peaks of NDIs were found at 3.65 Å for (R)−1 and (S)−1, whereas a slightly longer distance of 3.70 Å was observed for racemates. In addition, the RDFs indicated that interactions between NDIs exhibited the greatest strength, compared to interactions involving BNs or BN and NDI. Here, the stacking criterion was defined as a distance of 5.00 Å between the COG of the NDIs, corresponding to the distance of the first coordination shell. By applying the aforementioned stacking criterion, we distinguished stacked molecules into distinct clusters in the final configuration visualized with OVITO Pro.^[^ ^21^ ^]^ The assembly of (R)−1 and (S)−1 led to the formation of comparatively long structures, while the racemate resulted in shorter structures (Figure 1d,e), consistent with experimental results. The vertical distance between the two molecules exhibited a comparable value of 3.58 Å in both enantiomers and the racemate (Figure S6, Supporting Information). However, the probability of locating vertically at 3.58 Å was lower in the racemate due to the broader range of vertical distances, which tended to shift toward longer distances. Furthermore, an analysis of the dihedral angle distribution of stacked NDI planes demonstrated a predominantly coplanar alignment in (R)−1 and (S)−1, with averages of 7.05° and 7.14°, respectively. Conversely, racemates exhibited a more tilted arrangement at 8.15° (Figure 1f and Figure S7, Supporting Information).

The average properties of clusters in the assemblies were examined, with clusters defined as groups of two or more stacked molecules. As shown in Figure 1g, (R)‐1 and (S)‐1 exhibited greater counts of total and average stacked molecules in clusters compared to the racemate. The shorter π–π distance and smaller dihedral angle of enantiomers allowed favorable formation of longer clusters. These results were consistent with experimental self‐assembly, where enantiomers formed well‐defined 1D NWs and racemates formed ill‐defined NPs.

To investigate the thermodynamic properties of the assembly, structure optimization with density functional theory (DFT) calculations was performed to calculate the formation energy (Δ*E*_f_) of one stacking structure. The Δ*E*_f_ values were 0.03 eV smaller in racemate compared to (R)−1 and (S)−1 (Figure 1h and Figure S8, Supporting Information). The heterochiral assembly was more thermodynamically favorable than the homochiral assembly, supporting the occurrence of chiral self‐discrimination. Taken together, the simulation results supported that while heterochiral assembly is stable in terms of thermodynamics, it is structurally unfavorable due to the longer π‐π distance and tilted angular stacking of different enantiomers. As a consequence, it cannot form long crystalline 1D NWs like its homochiral counterpart, remaining as amorphous NPs. On the other hand, homochiral assembly adopts 1D NWs through regular and coplanar stacking.

### 2.3 Characterization of Materials

**Figure 2:**
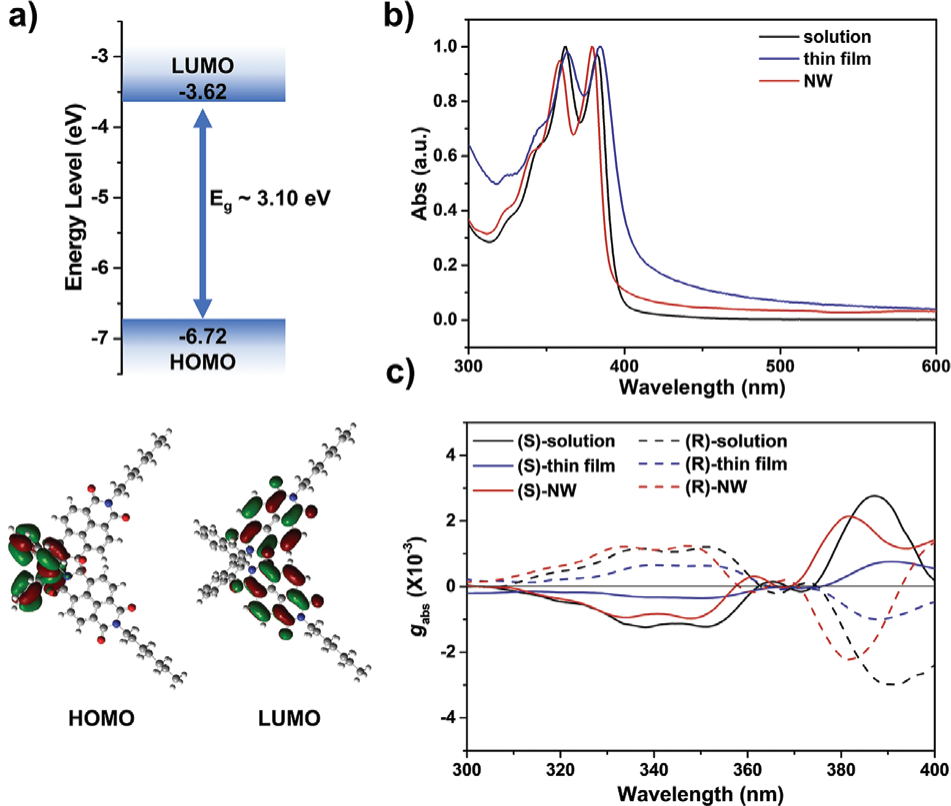
Electrochemical and chiroptical properties. a) Energy level and charge distribution. b) UV–vis spectra of (S)−1 in CHCl_3_ solution (1.0  ×  10^−5^ M), thin films, and NWs. c) Dissymmetry factor of absorption (*g*_abs_) of (R)−1 and (S)−1 in CHCl_3_ solution (1.0  ×  10^−5^ M), thin films, and NWs.

The electrochemical properties of (R)−1 and (S)−1 were investigated using cyclic voltammetry and DFT (**Figure** **2**a and Table S1, Supporting Information). The optical band gap was 3.10 eV, which was sufficiently wide to enable selective detection of UV. The experimental highest occupied molecular orbital (HOMO) and lowest unoccupied molecular orbital (LUMO) energies were −6.72 and −3.62 eV, respectively (Figure S9, Supporting Information). DFT calculations indicated that the HOMO primarily lies on BN, while the LUMO is located on the NDI, which is likely attributable to the electron‐donating nature of BN and the electron‐deficient character of the NDI π‐plane.^[^ ^22^ ^]^

The absorption spectra of (R)−1 and (S)−1 in chloroform solution, thin film, and NW showed similar features with three bands from 300 to 400 nm (Figure 2b). These bands corresponded to 0‐0, 0–1, and 0–2 transitions of electric dipole moment polarized along the long axis of the NDI core.^[^ ^23^ ^]^ It is noteworthy that absorption bands were not observed at wavelengths longer than 400 nm in all cases, making it applicable as a visible‐blind UV photodetector.

The chiroptical properties were then analyzed using CD spectroscopy. (R)−1 and (S)−1 showed a mirror image with *g*_abs_ values of around 10^−3^ (Figure 2c). Interestingly, there was no amplification of *g*_abs_ upon solid‐state aggregation, indicating that the chiroptical properties arose from intrinsic molecular chirality rather than supramolecular chirality. In the solid state, linear dichroism (LD) and linear birefringence (LB) can interfere with CD signals, leading to significant distortion of genuine *g*_abs_. Therefore, we conducted two experiments to confirm that the thin films did not have macroscopic anisotropic ordering (Figure S11, Supporting Information). First, we flipped the thin film samples by 180° along the vertical axis and compared the CD spectra. When asymmetric LD and LB terms are dominant in the thin film, it is known that the CD spectra will show a reverse sign when the sample is flipped.^[^ ^24^ ^]^ No sign inversion occurred in our case, indicating that the CD originated from intrinsic chirality, and not from the product of LD and LB. Second, we measured the CD spectra of the thin films by rotating the azimuthal angle of the film along the optical axis. The spectra were independent of the azimuthal angle, indicating the absence of anisotropic ordering.^[^ ^11^, ^25^ ^]^ In addition, the *g*_abs_ value at 385 nm remained consistent overall, regardless of the film thickness (Figure S12, Supporting Information).

### 2.4 Optoelectronic Performance

**Figure 3:**
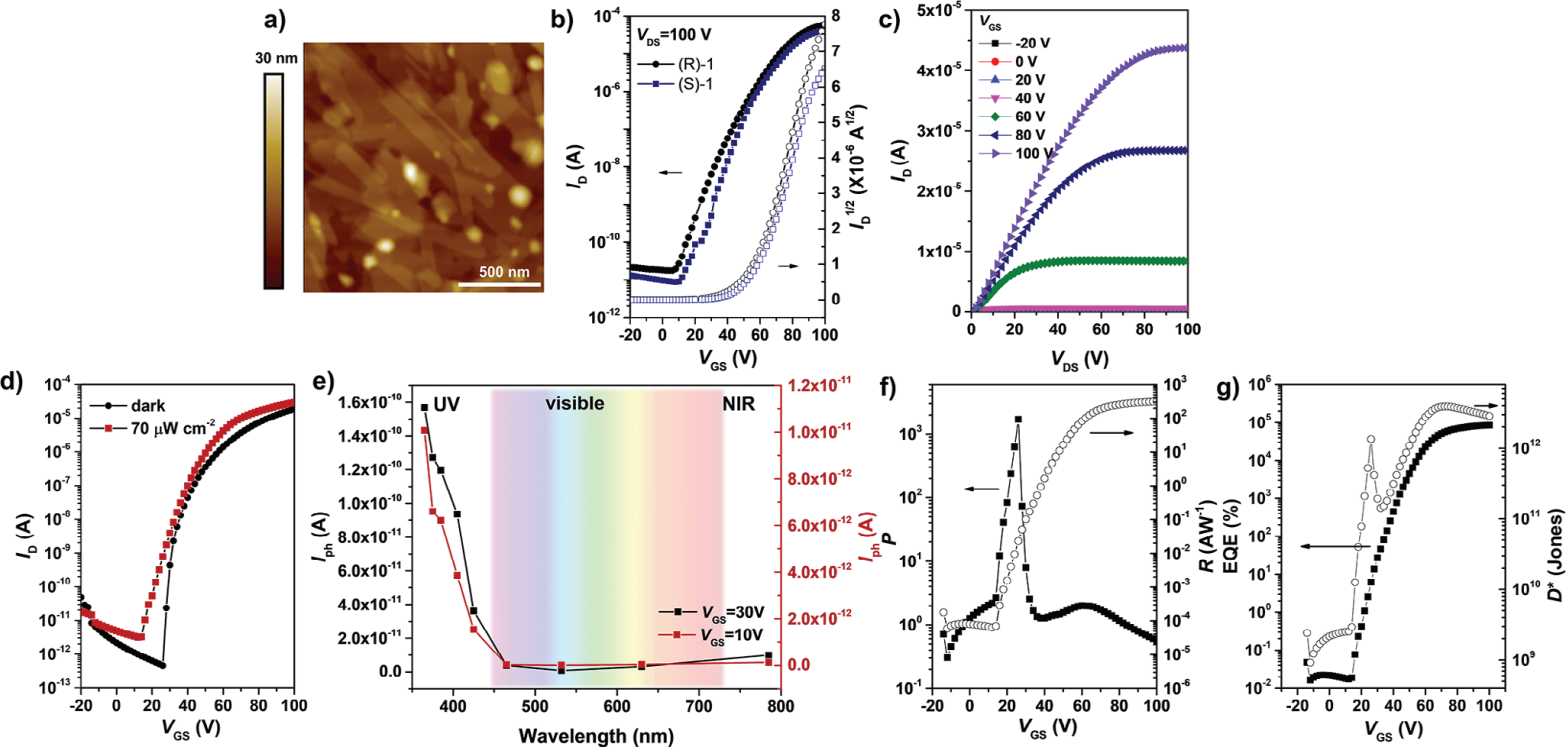
Optoelectronic performance. a) AFM height image of thermally deposited (S)−1 film at an optimized substrate temperature of 160 °C. b) Transfer characteristics of OFETs based on (R)−1 and (S)−1 films and c) output characteristics of OFETs based on (S)−1 films deposited at a substrate temperature of 160 °C. d) Transfer curves of (S)−1 films in the dark and under irradiation with UV light at a wavelength of 385 nm (70 µW cm^−2^). e) Photocurrent of (S)−1 films under monochromic light irradiation with different wavelengths from UV to NIR. f) Photocurrent/dark current ratio (*P*) and photoresponsivity (*R*). g) External quantum efficiency (EQE), and detectivity (*D* ^*^) of (S)−1 films under irradiation with UV light at a wavelength of 385 nm (70 µW cm^−2^).

Tapping‐mode atomic force microscopy (AFM) was performed to investigate the thin films of (S)−1 prepared on *n*‐octadecyltrimethoxysilane (OTS)‐modified SiO_2_/Si substrates. The thin films were fabricated through thermal evaporation under high vacuum (< 5 × 10^−6^ torr) with varying substrate temperature (*T*_s_) (**Figures** **3**a and S13, Supporting Information). As the *T*_s_ increased from room temperature to 160 °C, the grain size increased. (S)−1 formed dense granular grains at *T*_s_ of room temperatures, 75 and 125 °C. The grains were the largest at a *T*_s_ of 160 °C and formed a plate‐shaped microstructure with dense packing and fewer grain boundaries. However, when the *T*_s_ was increased to 180 °C, the grains were less connected due to severe desorption. Therefore, 160 °C was determined to be the optimal *T*_s_ for the fabrication of optoelectronic devices. This trend was consistent with previously reported thin films of thermally evaporated NDI derivatives.^[^ ^26^ ^]^

Whereas the thin film morphologies changed depending on *T*_s_, the chiroptical properties remained consistent. Temperature‐dependent CD analysis of (S)−1 films revealed almost constant *g*_abs_ values (Figure S14, Supporting Information). This suggests that the chiroptical properties of (R)−1 and (S)−1 result from the intrinsic molecular characteristics, and not from microstructural ordering.

To elucidate the electrical behavior of (R)−1 and (S)−1, organic field‐effect transistors (OFETs) with a bottom‐gate top‐contact (BGTC) configuration were fabricated. The experimental details regarding surface modification^[^ ^27^ ^]^ and OFET fabrication are presented in the Supporting Information. The transfer characteristics of OFETs fabricated with varying *T*_s_ are shown in Figure S15, Supporting Information and their electrical performance parameters are summarized in Table S2, Supporting Information. The device fabricated at a *T*_s_ of 160 °C exhibited the highest performance, characterized by enhanced average electron mobility and on/off ratio. The device performance improved as *T*_s_ increased from room temperature to 160 °C. However, a decline in performance was observed with further increases in *T*_s_. This trend was consistent with the trend in grain size observed by AFM.

The device fabricated at *T*_s_ of 160 °C exhibited high average electron mobility of 0.22 cm^2^ V^−1^ s^−1^ and an excellent on/off ratio of ≈10^6^. Typical transfer, output characteristics, and hysteresis test are shown in Figure 3b,c, and Figure S16, Supporting Information. The mobility was calculated by measuring the slope of the square root drain current along the gate voltage of the transfer curve in the saturation regime. It is remarkable that although the main backbone was significantly twisted for enhanced chirality, such efficient charge transport occurred, overcoming the dilemma of a trade‐off between chirality and charge transport.

Motivated by their selective absorption of light in the UV region, we investigated the photoresponse performance of OPTs. The OPT devices were fabricated in a BGTC configuration, using the same conditions as described for the aforementioned OFETs. First, we investigated the transfer characteristics of the OPT devices in the dark and under monochromatic light illumination at 385 nm with an intensity of 70 µW cm^−2^ (Figure 3d). The drain current increased upon illumination, and the threshold voltage (*V*_T_) showed a negative shift, indicating easier turn‐on under *n*‐channel operation. The increase in drain current and the considerable shift in *V*_T_ were due to the photogenerated charge carriers and reduced trap sites.^[^ ^11^, ^28^ ^]^ In addition, the device was illuminated with monochromatic light with different wavelengths from UV to near‐infrared to determine the wavelength selectivity. The photocurrent (*I*_ph_) was estimated under illumination with different types of light in both photoconductive mode (*V*_GS_ = 10 V) and phototransistor mode (*V*_GS_ = 30 V) (Figure 3e and Figure S19, Supporting Information). Specifically, for illumination light with wavelengths longer than 450 nm, the relative photocurrent of the given wavelength to UV (*I*_ph, given wavelength_/*I*_ph, 365 nm_) was less than 1.29% and 6.31% for *V*_GS_ = 10 V and *V*_GS_ = 30 V, respectively. This implied that the OPT can respond selectively to the narrowband UV light, enabling its potential use as a miniature visible‐blind UV photodetector without the need for expensive and bulky optical filters.^[^ ^26^, ^29^ ^]^ The UV photoresponse was further quantified with parameters such as photocurrent/dark current ratio (*P*), photoresponsivity (*R*), external quantum efficiency (EQE), and detectivity (*D* ^*^) (Figure 3f,g, Figure S18, and Table S3, Supporting Information). The estimation of these photoparameters is described in detail in the Supporting Information. The maximum values obtained for *P*, *R*, EQE, and *D* ^*^ were 1.7  ×  10^3^, 314 A W^−1^, 8.5  ×  10^4^%, and 3.9  ×  10^12^ Jones, respectively for (S)−1. In particular, *D* ^*^ values calculated from shot noise under dark conditions^[^ ^30^ ^]^ are among the most important factors in OPTs. The fabricated OPTs showed high *D* ^*^, comparable to planar NDI derivatives without chirality.^[^ ^26^ ^]^ The optoelectronic performance was outstanding compared to other chiral organic phototransistors reported to date, due to the efficient exciton dissociation and charge transport (Table S4, Supporting Information). This study demonstrated the enhanced performance achieved by rationally linking BN and NDIs to achieve superior *n*‐channel charge transport characteristics, an area that has not been explored in as much detail as its *p*‐channel counterparts for chiral small molecules. Fabricated devices exhibited both high electron mobility and high detectivity, despite their distorted structures. Importantly, the demonstration of visible‐blind UV selective characteristics, an aspect that has been largely unexplored, expands the potential applications, including areas such as biological diagnostics, quantum communication, and military utilization.

### 2.5 Selective CPL Sensing

**Figure 4:**
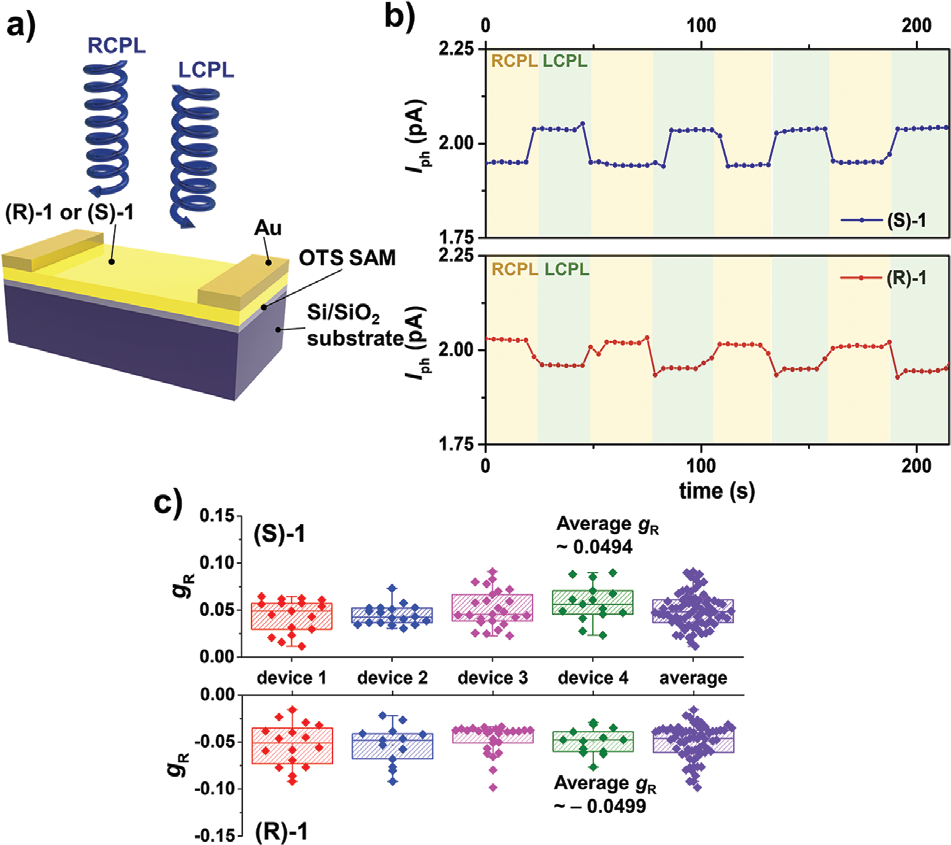
Selective CPL detection. a) Schematic diagram of device structure and CPL detection setup. b) Real‐time photocurrent of thermally deposited (S)−1 and (R)−1 OPT devices under 385 nm CPL irradiation c) Statistical results of *g*_R_ of four (S)−1 and (R)−1 devices under 385 nm CPL irradiation.

According to the chiroptical properties described earlier, (R)−1 and (S)−1 have the potential to selectively distinguish between two polarized states of UV light. Therefore, we conducted further investigations to determine the ability of (R)−1 and (S)−1 to sense CPL. CPL was generated using a 385 nm light source that passed sequentially through a linear polarizer and a quarter‐wave plate (**Figure** **4**a). Figure 4b displays the real‐time photocurrent of (R)−1 and (S)−1 OPTs when exposed repeatedly to right‐handed CPL (RCPL) and left‐handed CPL (LCPL). This indicates that differential absorption of CPL has led to the successful differential generation of photo‐induced carriers, and thus results in differential electric signals.^[^ ^12c^ ^]^ (R)−1‐based OPTs showed higher *I*_ph_ when RCPL was illuminated, whereas (S)−1‐based OPTs showed higher *I*_ph_ when LCPL was illuminated. The selective sensing of CPL is due to the intrinsic molecular chirality induced by the BN moiety, as discussed earlier. The selective response to CPL can be quantified by the dissymmetry factor of responsivity (*g*_R_), using the following equation,^[^ ^31^ ^]^ where *R*_L_ and *R*_R_ indicate photoresponsivity to LCPL and RCPL, respectively.

(2) $$g_{R}=2\left(\frac{R_{L}-R_{R}}{R_{L}+R_{R}}\right)$$

Figure 4c summarizes the *g*_R_ values calculated from over 60 points in four different devices. The average *g*_R_ values were 0.0494 ± 0.0180 for (S)−1 and –0.0499 ± 0.0183 for (R)−1. The *g*_R_ values were larger than the *g*_abs_ values, which is consistent with other organic CPL detectors. In the literature, it has been suggested that the combined impact of the photomultiplication by the applied gate bias and the spin‐dependent transport and collection of carriers by the optical selection leads to a synergistic increase in *g*_R_ compared to *g*_abs_.^[^ ^11^, ^32^ ^]^ These remarkable results regarding CPL sensing offer opportunities for the development of visible‐blind UV‐selective CPL detectors.

## 3 Conclusion

In summary, we have synthesized a novel *n*‐type chiral small molecule that links two NDIs and a BN and investigated the charge transport and UV CPL detecting properties in OPT devices. The self‐assembly process yielded NWs for pure enantiomers, whereas a gradual decrease in enantiomeric excess led to the formation of NPs. Computational analysis further supported the observed chiral self‐discrimination behavior. Additionally, we fabricated a phototransistor based on thermally evaporated thin films. Notably, the OPTs achieved a relatively high electron mobility of 0.22 cm^2^ V^−1^ s^−1^, accompanied by a *g*_R_ value of ≈0.05 in sensing CPL.

Our results demonstrate the potential of small molecule‐based organic CPL detectors. By utilizing well‐accumulated knowledge of BN derivatives in synthetic chemistry and integrating it into chiral optoelectronics, we envision the possibility of designing customized molecules with desired properties. For instance, by incorporating functional groups onto BN, we can readily manipulate the dihedral angle, which influences molecular distortion and CPL absorption. Additionally, the narrowband absorption in the UV region demonstrated in this study opens up new possibilities for various applications, including quantum communication or biological detection using UV CPL without interference from room lighting.

## Conflict of Interest

The authors declare no conflict of interest.

## Supporting information

Supporting Information

## Data Availability

The data that support the findings of this study are available from the corresponding author upon reasonable request.
