# Supplementary material for: Axially Chiral Organic Semiconductors for Visible‐Blind UV‐Selective Circularly Polarized Light Detection
Source: Adv Sci (Weinh). 2024 Feb 4;11(14):2308262. doi: 10.1002/advs.202308262 (PMC11005684; doi:10.1002/advs.202308262)
Supplement: Supplementary file 1 — Supporting Information [file ADVS-11-2308262-s001.pdf]

ADVANCED SCIENCE
Open Access

Supporting Information

for *Adv. Sci.*, DOI 10.1002/advs.202308262

Axially Chiral Organic Semiconductors for Visible-Blind UV-Selective Circularly Polarized Light Detection

Yejin Kwon, Je-Yeon Jung, Won Bo Lee and Joon Hak Oh**

Supporting Information

Axially Chiral Organic Semiconductors for Visible-Blind UV-Selective Circularly Polarized Light Detection

Yejin Kwon, Je-Yeon Jung, Won Bo Lee, and Joon Hak Oh**

Table of Contents

General procedures and methods	S3
Experimental procedures	S4~S6
Estimation of Optoelectrical Properties	S7
Synthetic procedures and structural data	S7~S11
TEM and SEM images	S12
OM images and CD spectra with varying enantiomeric excess (ee)	S13
MD and DFT simulation results	S14~S15
Cyclic voltammograms and energy levels	S16
TGA and DSC thermograms	S17
Chiroptical properties of thin films	S18
AFM topography images and CD spectra of thin films with varying substrate temperature	S19
OFET performance of **(S)-1** films with varying substrate temperature	S20
Transfer curves of **(R)-1** and **(S)-1** films with forward and reverse sweeps and in the lienar regime	S21
Optoelectronic performance of **(R)-1** and **(S)-1** based OPTs.	S22
Photoresponse of OPT devices under monochromic light with different wavelength	S23
Rise and fall time estimation	S23
Transfer curves of OPTs in dark or under CPL illumination	S24
Comparison of this work and other recently published chiral organic small molecule OP Ts	S25
References	S26

General Procedures and Methods

The starting material, naphthalene-1,4,5,8-tetracarboxylic dianhydride, was purchased from Alfa Aesar, while all other reagents were obtained from Sigma Aldrich. All solvents were used as ACS grade. ^{1}H NMR spectra were recorded on an Avance III (Bruker, Germany) spectrometer using $CDCl_3$ as the solvent. MALDI-TOF MS spectra were acquired using a Voyager DE-STR (Applied Biosystems, USA) instrument. The absorption spectra were measured using a Cary 5000 UV−vis−NIR spectrophotometer (Agilent, USA). CD results were obtained using a J-815 spectropolarimeter (JASCO, Japan). For CD measurements, the nanowire or nanoparticle dispersed in methylcyclohexane was tested to eliminate the anisotropic property and the thin films were thermally deposited on transparent quartz plates. The optical rotation was measured using P-2000 Polarimeter (Jasco, Japan) at 20 °C with a WI light source at 589 nm. Thermal properties were investigated using Pyris 6 (Perkin Elmer, USA) TGA and DSC 25 (TA instruments, USA). SEM images were captured using a JSM-7800F Prime (JEOL Ltd, Japan) and TEM image were determined with a JEM-F200 (JEOL Ltd, Japan) operated at 200 kV. AFM image was earned using NX-10 (Park Systems, Korea). DFT calculations were performed using the Gaussian 09 package with the nonlocal hybrid Becke three-parameter Lee-Yang-Parr (B3LYP) function and the 6-31G basis set to elucidate the HOMO and LUMO levels after optimizing the geometry of **(R)-1** and **(S)-1**.

Experimental Procedures

OFET fabrication. OFETs based on thin films of **(S)-1** and **(R)-1** were fabricated using heavily *n*-doped (100) silicon wafers with 300 nm thick SiO_2 (C_i = 11.5 nF cm^{-2}). Wafers were cleaned with a piranha solution for 30 min and underwent UV-ozone treatment. The wafer surface was then treated with *n*-octadecyltirmethoxysilane (OTS) to form a self-assembled monolayer. 3 mM OTS solution in trichloroethylene was spin-coated at 500 rpm for 5 s, at 1500 rpm for 30 s, and at 500 rpm for 5 s. Then, the wafer was exposed to ammonia vapor overnight in a glass desiccator. Synthesized **(R)-1** and **(S)-1** was deposited (40 nm thickness) on the wafer by thermal evaporator under different substrate temperatures. Thermal evaporation was conducted at a crucible temperature of 230–260 ^{o}C in a rate of 0.1 Å s^{-1} under ultrahigh vacuum condition (< 5 × 10^{-6} torr). Gold electrodes (40 nm thickness) were thermally evaporated using shadow masks to form source and drain electrodes with a channel length (*L*) of 50 μm and a channel width (*W*) of 1000 μm.

Optoelectronic measurements. The electrical performance of OFETs was measured in N_2 environment using Kiethley 4200-SCS semiconductor parametric analyzer. Photoresponse characteristics were measured inside a vacuum chamber and monochromic 385nm LED (Thorlabs, M385L3) was used. For testing the spectral response, monochromic light was generated using a 300 W Xenon lamp and Oriel Cornerstone 130 monochromator with dual gatings. The circularly polarized light was generated using a linear polarizer and a quarter-wave plate (Thorlabs, AQWP05M-340). The generated light was calibrated by placing the conventional Si photodetector in the same position with the devices.

Computational analysis (Molecular Dynamics Simulations). Three systems were simulated, named R_{sys}, S_{sys} and $Race_{sys}$. Each system consisted of 24 molecules of **(R)-1**, 24 molecules of **(S)-1** and 12 molecules of both **(R)-1** and **(S)-1**, with 2400 molecules of MCH solvent. Initial box size of all systems was 9.5×9.5×9.5 nm. During the process of constructing the initial configuration of R_{sys}, a 2×3×4 array of 24 molecules of **(R)-1** was placed in parallel. The COG distances between the parallel NDIs of each molecule along the x, y, and z axes were set to 2.0 nm, 2.5 nm, and 1.5 nm. These distances are significantly longer than the stacking distance.[S1] The initial configuration of S_{sys} consisted of 4×3×2 array of 24 molecules of **(S)-1**, with distances of 1.5 nm, 2.0 nm, and 2.5 nm along the axes. For $Race_{sys}$, a 4×3×2 array of 12 molecules of **(R)-1** and 12 molecules of **(S)-1**, arranged in parallel. To prevent the adjacent

placement of same molecule type, **(R)-1** and **(S)-1** were alternately placed. A solvent was randomly placed in the void to prevent atom overlapping.

All the systems and simulations were performed under 1 bar and 363 K, which is below the boiling point of MCH solvent. The simulation process started with energy minimization with steepest descent method. Subsequently, a 1 ns NPT run was conducted with Velocity Rescaling (v-rescale) thermostat and Berendsen barostat to rapidly approach density values near equilibrium. A 5 ns NPT run was carried out with Nose-Hoover thermostat and Parrinello-Rahman barostat was followed by a 150 ns NVT run with Nose-Hoover thermostat to achieve an equilibrium state. Additionally, 20ns production run under NVT ensemble was performed and used in analysis process.

All molecular dynamics (MD) simulations were conducted using the GROMACS molecular dynamics simulation package.[S2] Forcefield parameters for **(R)-1**, **(S)-1**, MCH were generated with PolyParGen,[S3] based on the OPLS-AA forcefield.[S4] Furthermore, structural modifications were applied to clarify the chiral structures using the Avogadro 2 program.[S5] In the simulations, cutoff distance of 1.0 nm for an electrostatic and Van der Waals potential was set, and periodic boundary conditions were applied. For handling long-range electrostatic potentials, Particle Mesh Ewald method[S6] was applied. During the production run, simulation time step was 1fs and trajectory data was saved at intervals of 1 ps.

Computational analysis (Density Functional Theory), Density functional theory (DFT) calculations for determining the formation energy of homochiral and heterochiral stacking were performed using the Vienna Ab initio Simulation Package (VASP)[S7] with the Projector Augmented Wave (PAW) method.[S8]

The initial structures of **(R)-1**, **(S)-1**, and the racemate were obtained from the final configuration of the MD simulations and modified by tilting two molecules about 74 to 77 degrees to construct stacking structure using ASE (The Atomic Simulation Environment).[S9] The structure optimization at the gamma point with 520 eV of a plane wave energy cutoff. The Perdew-Burke-Ernzerhof (PBE) functional[S10] and van der Waals dispersion correction were applied to calculate energy of stable structures. The calculations were continued until the energy difference of the total and the band structure energy became smaller than 10^{-6} eV, with a maximum force criterion for convergence set to 0.05 eV $Å^{-1}$. Mixing parameters from Kerker mixing scheme[S11] of A, and B were set to 0.3 and 0.5.

For the initial configuration, systems were constructed with 2 molecules of either **(R)-1** and **(S)-1** of the same type of different types. These two molecules were positioned at a minimum

distance which is about 3.85 ± 0.2 Å from one of the inner atoms constituting the COG of within the NDIs, which is shorter than the stacking criteria distance, in order to mimic the stacking structure without atomic overlap. To focus on a single pure stacking interaction between NDIs, a vacuum was set along all axes more than 10 Å. Additionally, calculations were performed for systems consisting of a single molecule of **(R)-1** and **(S)-1** under the same conditions above to determine the energy difference between those two molecule systems, which represents the formation energy.

For heterochiral stacking formation energy,

$$\Delta E_{f,\ hetero} = \frac{\left(E_{hetero} - E_{(\mathrm{R})\text{-}1} - E_{(\mathrm{R})\text{-}1}\right)}{2} \tag{S1}$$

For homochiral stacking formation energy,

$$\Delta E_{f,\ homo} = \frac{\left(E_{homo} - 2E_{(R)\text{-}1\ (S)\text{-}1}\right)}{2} \tag{S2}$$

Cyclic Voltammetry. Cyclic voltammetric measurements were carried out using a three-electrode cell consisting of a polished 2 mm glassy carbon as the working electrode, Pt as the counter electrode, and $Ag/AgNO_3$ as the reference electrode. Solutions in chloroform were prepared with concentrations of 1.0×10^{-3} M for **(R)-1** and **(S)-1**, and 0.10 M for the supporting electrolyte, tetrabutylammonium hexafluorophosphate ($TBAPF_6$). To determine the LUMO levels relative to the vacuum level, the redox data were standardized to the ferrocene/ferrocenium couple with a calculated absolute energy of −4.8 eV, using the following equation;

$$\mathrm{HOMO\ (eV)} = -(E_{(\mathrm{ox})}{}^{\mathrm{onset}} - E_{(\mathrm{ferrocene})}{}^{\mathrm{onset}} + 4.8) \tag{S3}$$

Estimation of Optoelectrical Properties

To explore the photosensitivity of organic phototransistors, the transfer characteristics were analyzed under light irradiation to determine the photoresponsivity (R) and the ratio of photocurrent to dark current (P). The values for R and P were determined using the following equations:

$$R = \frac{I_{ph}}{P_{inc}} = \frac{I_{light} - I_{dark}}{P_{inc}} \quad \text{(S4)}$$

$$P = \frac{I_{light} - I_{dark}}{I_{dark}} \quad \text{(S5)}$$

where I_{ph} is the photocurrent, P_{inc} the incident illumination power on the channel of the device, I_{light} the drain current under illumination, and I_{dark} the drain current in the dark, respectively.

Furthermore, the external quantum efficiency (EQE) (η) of OPTs was calculated. This quantity is expressed as the ratio of the number of photogenerated carriers that effectively increase the drain current to the number of photons that are incident on the area of the OPT channel. This can be determined using the following equation.

$$\eta = \frac{(I_{light} - I_{dark})hc}{eP_{int}A\lambda_{peak}} \quad \text{(S6)}$$

where h is the plank constant, c the speed of light, e the fundamental unit of charge, P_{int} the incident power density, A the area of the transistor channel, and λ_{peak} the peak wavelength of the incident light, respectively.

The concept of detectivity typically refers to the minimum detectable signal, which allows the comparison of photodetector devices with varying configurations and sizes. In this study, D^* was determined by applying equations (S7) and (S8).

$$D^* = \frac{\sqrt{A}}{NEP} \quad \text{(S7)}$$

$$NEP = \frac{\sqrt{I_n^2}}{R\sqrt{\Delta f}} \quad \text{(S8)}$$

In these equations, A is the photodetector active area, NEP the noise equivalent power, and I_n^2 the measured noise current, and Δf the bandwidth. If the major limit to detectivity is shot noise from the drain current under dark conditions, D^* can be simplified as equation (S9).

$$D^* = \frac{R}{\sqrt{\frac{2eI_{dark}}{A}}} \quad \text{(S9)}$$

The rise time is defined to be the time required for the current to increase from 10% to 90% of the peak value after light illumination, whereas the fall time is estimated to be the time required for the current to decrease from 90% to 10% of the maximum value.

Synthetic procedures and structural data

Scheme S1.

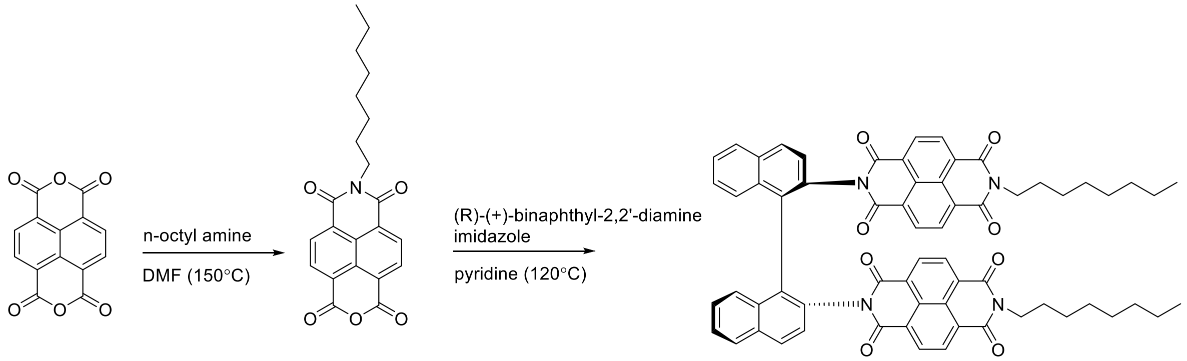

2 **(R)-1**

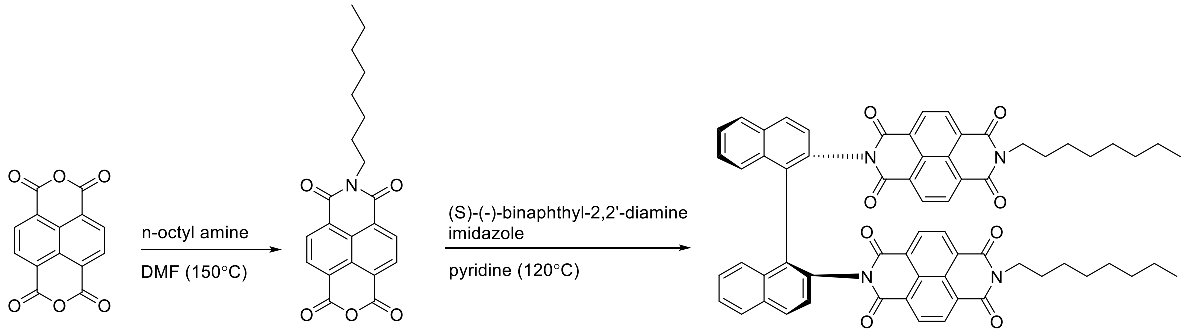

2 **(S)-1**

Scheme S1. Synthetic routes to **(R)-1** and **(S)-1**. n-octyl amine and binpahthyl-2,2’-dimamine was reacted successively with naphthalene-1,4,5,8-tetracarboxylic dianhydride.

7-octyl-1H-isochromeno[6,5,4-def]isoquinoline-1,3,6,8(7H)-tetraone ***(2)*** [S12]

Naphthalene-1,4,5,8-tetracarboxylic dianhydride (5.36 g, 20.0 mmol, 4 equiv.) was dissolved in 30 mL dry DMF under N_2 and was stirred for two hours. To the mixture, *n*-octyl amine (0.83 mL, 5.00 mmol, 1 equiv.) was added dropwise and stirred at 150 °C overnight. After the completion of reaction was monitored using TLC, the reaction mixture was cooled to 0 °C. The resulting precipitate was filtered and dissolved in dichloromethane. The compound was washed with brine and dried over $MgSO_4$. **2** was used directly for next reaction without further purification. Only a small amount was purified by silica gel column chromatography for characterization purpose. ^{1}H NMR (400 MHz, $CDCl_3$) δ= 9.00 – 8.65 (m, 3H), 4.35 – 3.97 (m, 2H), 1.82 – 1.66 (m, 2H), 1.44 – 1.09 (m, 8H), 0.88 (t, *J* = 6.4 Hz, 3H). MALDI-TOF MS (m/z) [M^+] calculated for: 379.14197, found: 379.17539.

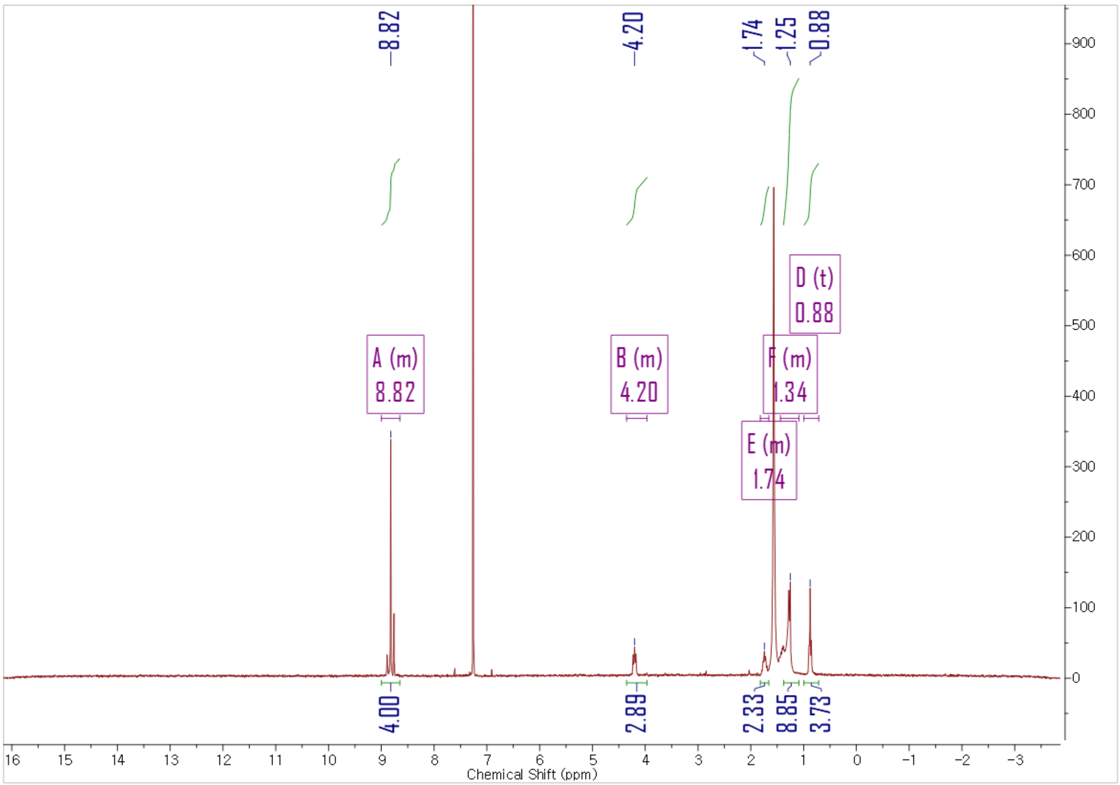

(R)-7,7'-([1,1'-binaphthalene]-2,2'-diyl)bis(2-octylbenzo[lmn][3,8]phenanthroline-1,3,6,8(2H,7H)-tetraone) ***((R)-1)***

A solution of **2** (0.5085 g, 1.2795 mmol, 2.5 equiv.), (R)-1,1′-binaphthyl-2,2′-diamine (0.1455 g, 0.5118 mmol, 1 equiv.) and imidazole (0.3484 g, 5.1179 mmol, 10 equiv.) in 20 mL pyridine was stirred at 120 °C under N_2 overnight. After the completion of reaction was monitored using TLC, the mixture was poured to 50 mL 1N HCl and stirred for a while. The mixture was filtered and washed with water and methanol several times. The solid residue was dissolved in dichloromethane and dried over $MgSO_4$ The crude product was purified by silica gel column chromatography with hexane:EA=3:1 as eluent. Isolated yield=0.1157 g (22 %) as a yellow solid. m.p.=296.2 °C $[\alpha]_{589nm}^{20°C} = -16.23°$ (*c*=1 in THF) ^{1}H NMR (400 MHz, $CDCl_3$) δ= 8.80 (d, *J* = 7.6 Hz, 2H), 8.56 (d, *J* = 7.6 Hz, 2H), 8.20 (d, *J* = 7.6 Hz, 2H), 8.14 (d, *J* = 7.6 Hz, 2H), 7.98 – 7.88 (m, 4H), 7.58 (ddd, *J* = 8.1, 5.7, 2.3 Hz, 2H), 7.43 – 7.37 (m, 4H), 7.22 (d, *J* = 8.7 Hz, 2H), 4.20 (t, 4H), 1.79 – 1.67 (m, 4H), 1.51 – 1.18 (m, 40H), 0.88 (t, *J* = 6.9 Hz, 6H). MALDI-TOF MS (m/z) $[M+H]^+$ calculated for: 1007.39416, found: 1007.41132. Elemental analysis for $C_{64}H_{54}N_4O_8$: Calcd C 76.32, H 5.40, N 5.56, Found: C 74.64, H 5.49, N 5.83.

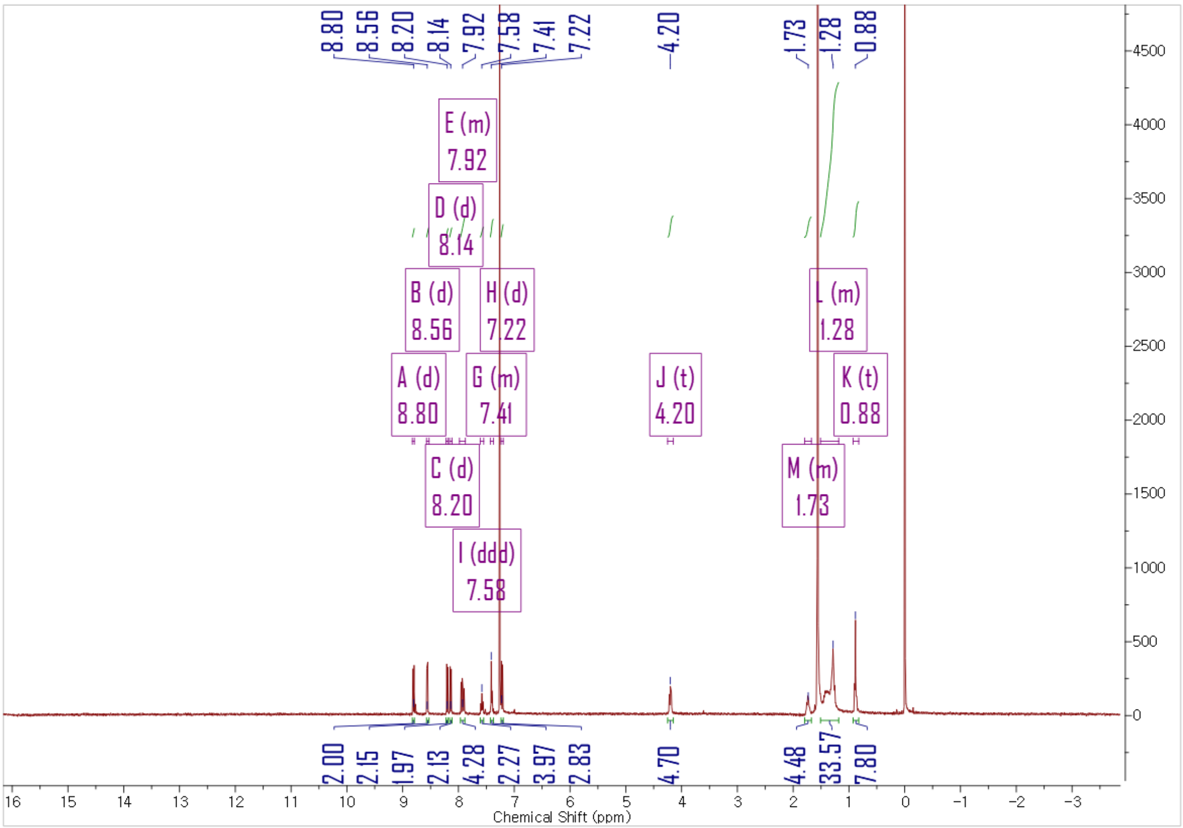

(S)-7,7'-([1,1'-binaphthalene]-2,2'-diyl)bis(2-octylbenzo[lmn][3,8]phenanthroline-1,3,6,8(2H,7H)-tetraone) **((S)-1)**

A solution of **2** (0.5542 g, 1.3945 mmol, 2.5 equiv), (S)-1,1'-binaphthyl-2,2'-diamine (0.1586 g, 0.5578 mmol, 1 equiv) and imidazole (0.3797 g, 5.5778 mmol, 10 equiv) in 20 mL pyridine was stirred at 120 °C under N_2 overnight. The synthetic procedure is similar as described for **(R)-1**. Isolated yield=0.0947 g (17 %) as a yellow solid. m.p.=294.7 °C $[\alpha]_{589nm}^{20℃} = +14.16°$ (*c*=1 in THF). ^{1}H NMR (400 MHz, $CDCl_3$) δ= 8.81 (d, *J* = 7.6 Hz, 2H), 8.56 (d, *J* = 7.6 Hz, 2H), 8.20 (d, *J* = 7.6 Hz, 2H), 8.14 (d, *J* = 7.6 Hz, 2H), 7.98 – 7.87 (m, 4H), 7.58 (ddd, *J* = 8.1, 5.4, 2.5 Hz, 2H), 7.45 – 7.35 (m, 4H), 7.22 (d, *J* = 8.7 Hz, 2H), 4.20 (t, 4H), 1.82 – 1.66 (m, 4H), 1.51 – 1.17 (m, 40H), 0.88 (t, *J* = 6.8 Hz, 6H). MALDI-TOF MS (m/z) $[M+H]^+$ calculated for: 1007.39416, found: 1007.34876. Elemental analysis for $C_{64}H_{54}N_4O_8$: Calcd C 76.32, H 5.40, N 5.56, Found: C 73.47, H 5.36, N 6.04.

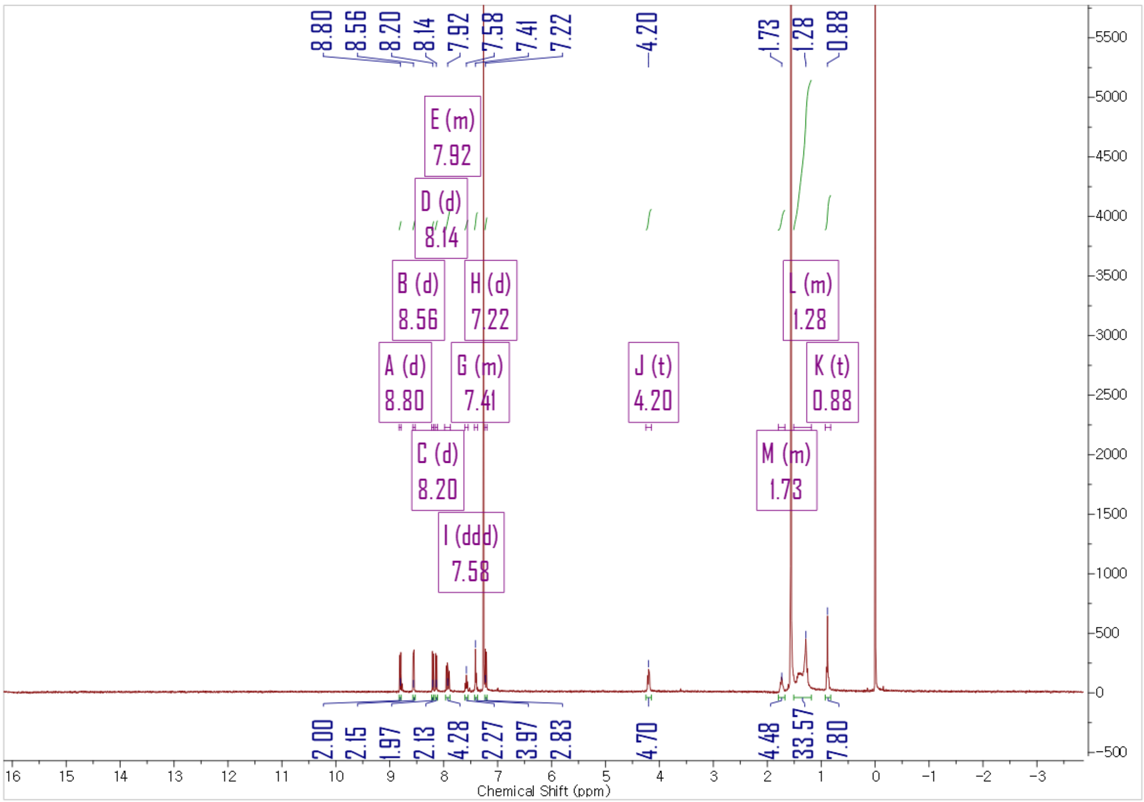

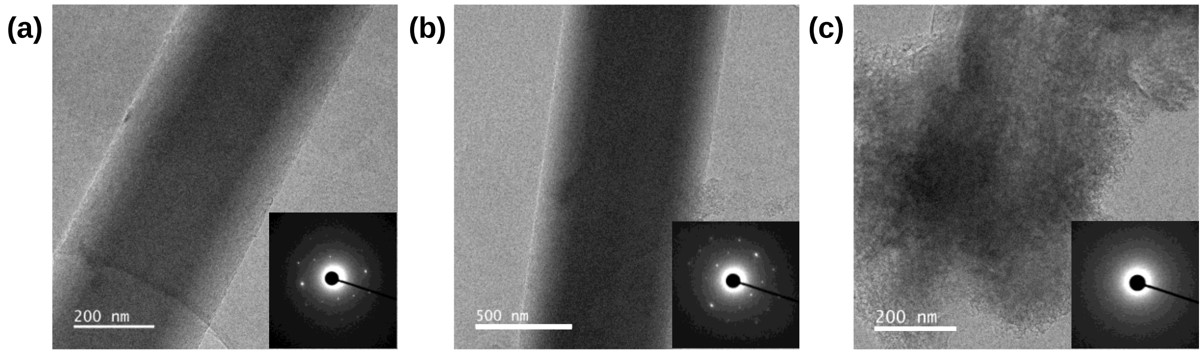

Figure S1. TEM image and SAED pattern (inset) of self-assembled structures of (a) **(R)-1,** (b) **(S)-1** and (c) racemate.

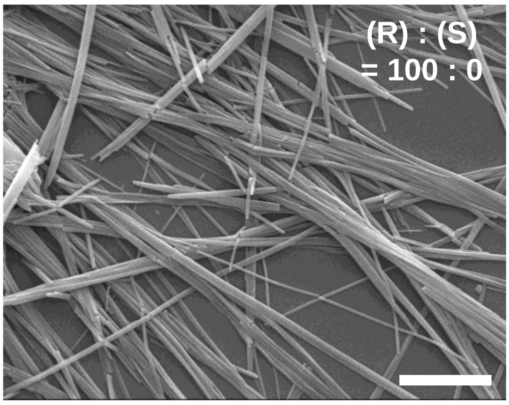

Figure S2. SEM images of **(R)-1** self-assembled NW structures (Scale bars, 3 μm).

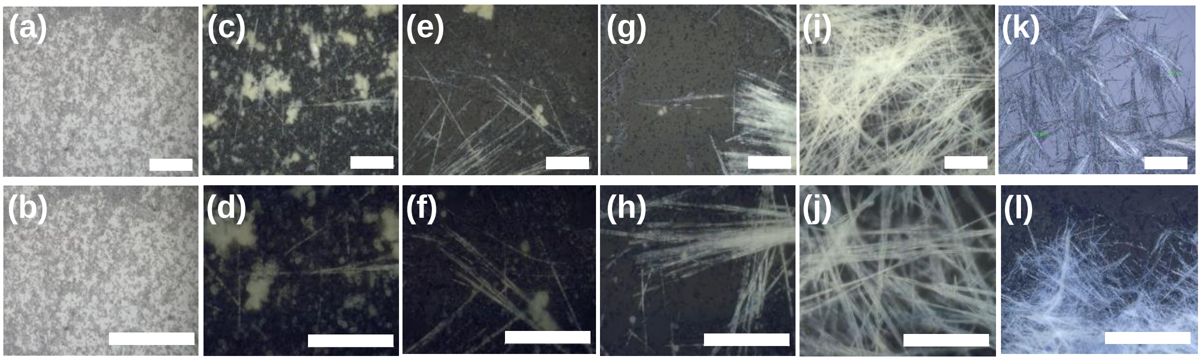

Figure S3. Optical microscope images of coassemblies in different **(R)-1**:**(S)-1** ratio (a), (b) 50:50, (c), (d) 40:60, (e), (f) 30:70, (g), (h) 20:80, (i), (j) 10:90, (k), (l) 0:100 (Scale bars, 50 μm).

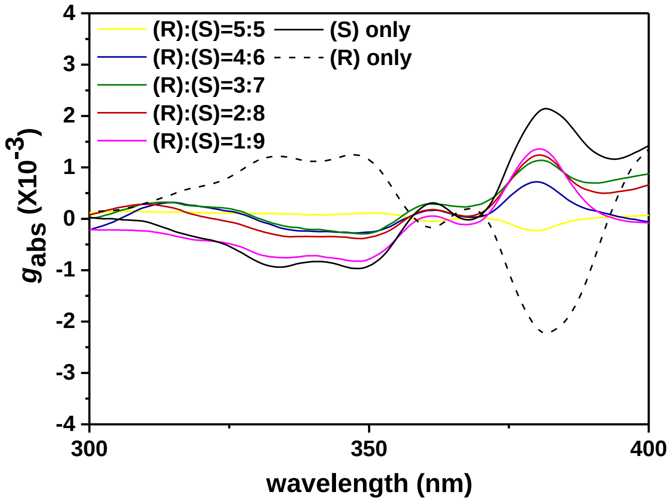

Figure S4. g_{abs} spectra of coassemblies in different **(R)-1**:**(S)-1** ratio.

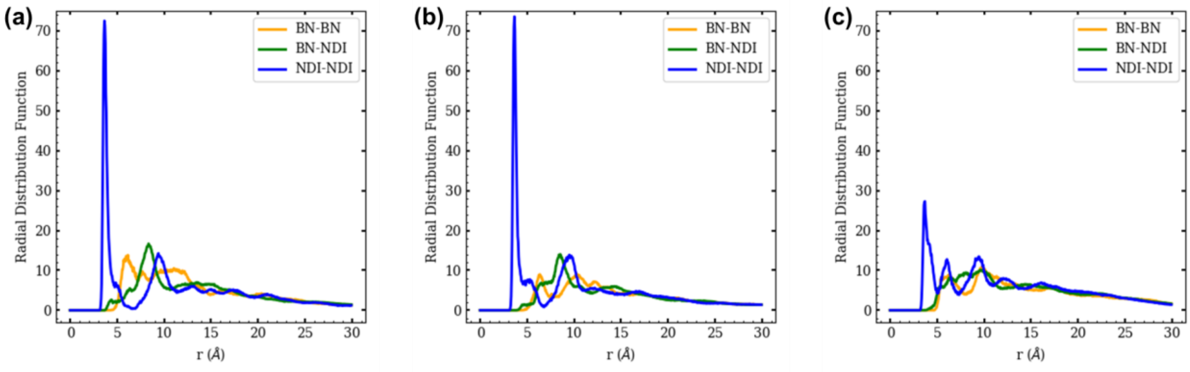

Figure S5. Racial distribution functions. (a) **(R)-1** (b) **(S)-1** (c) Racemic. The yellow line represents the RDFs of BN and BN, the green line represents NDI and BN, and the blue line represents NDI and NDI.

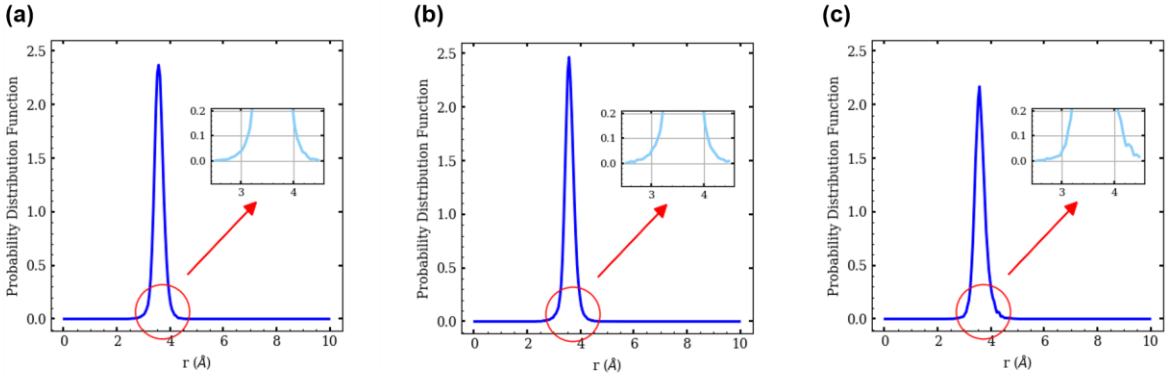

Figure S6. Vertical distance between two stacked NDIs. (a) **(R)-1** (b) **(S)-1** (c) Racemic.

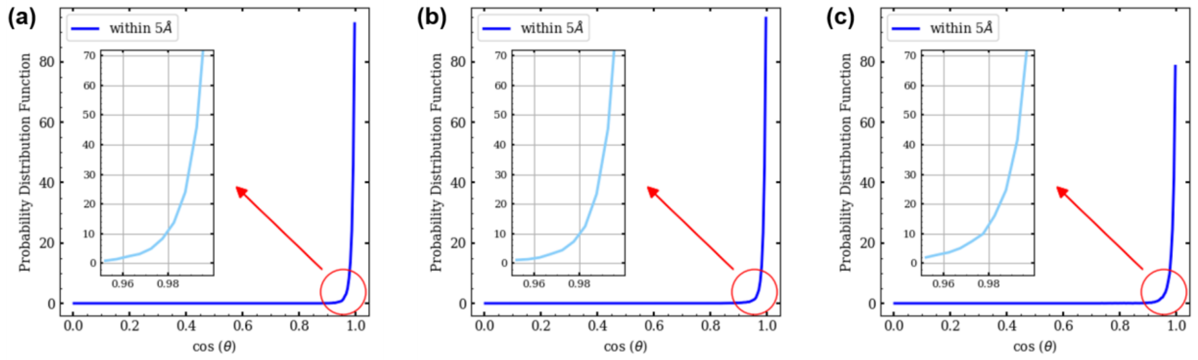

Figure S7. Probability distribution function of facing angle difference between two NDIs. (a) **(R)-1** (b) **(S)-1** (c) Racemic.

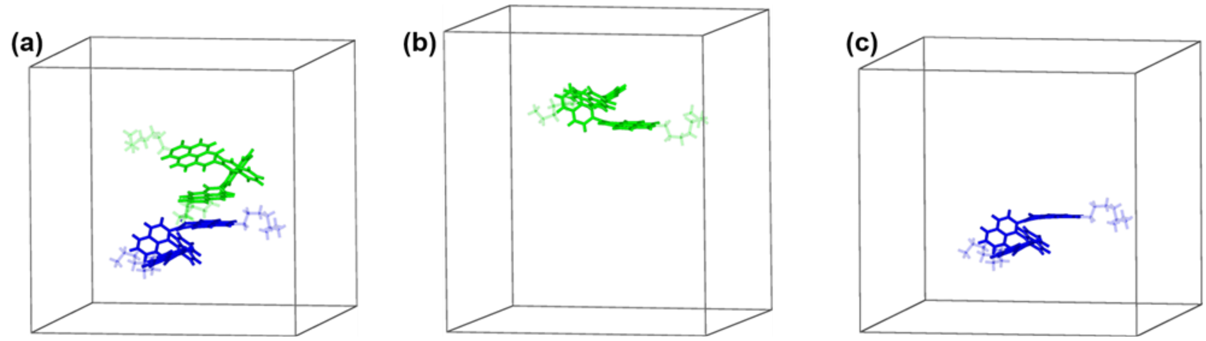

Figure S8. Final structure obtained from structure optimization. (a) Racemate (b) **(R)-1** (c) **(S)-1**.

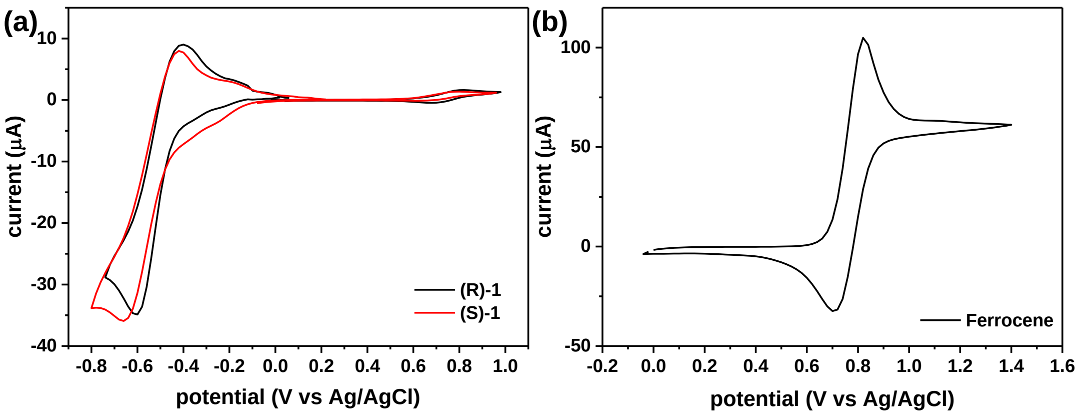

Figure S9. Cyclic voltammograms (a) **(R)-1** and **(S)-1** (b) ferrocene.

Table S1. Energy level of **(R)-1** and **(S)-1** from experimental[a] measurement and DFT calculation

Material	Molecular Orbital	Energy Level (eV)	
		**experimental**	**DFT**
**(R)-1**	LUMO	-3.62	-3.75
	HOMO	-6.72	-6.17
**(S)-1**	LUMO	-3.63	-3.75
	HOMO	-6.73	-6.17

[a] The LUMO levels were measured by cyclic voltammetry. The HOMO levels were calculated using the optical band gap of UV-vis spectra

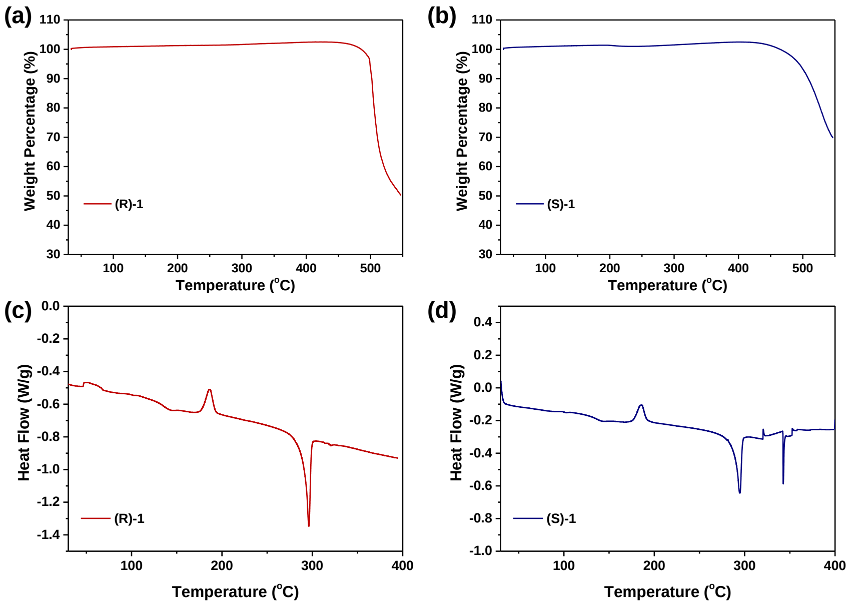

Figure S10. TGA thermograms of (a) **(R)-1** and (b) **(S)-1** and DSC thermograms of (c) **(R)-1** and (d) **(S)-1**. The first endothermic peak corresponds to the melting point. Above 450 °C, **(R)-1** and **(S)-1** were decomposed.

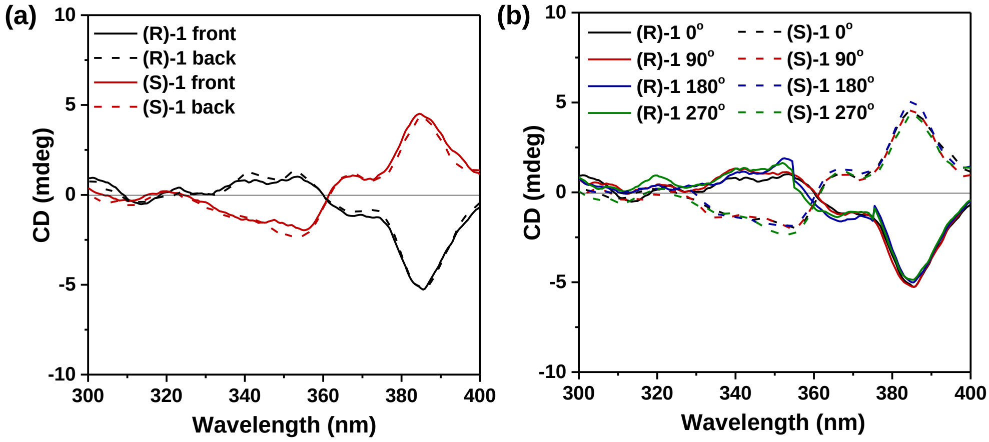

Figure S11. CD spectra of **(R)-1** and **(S)-1** films measured with (a) sample flipping and (b) sample rotation.

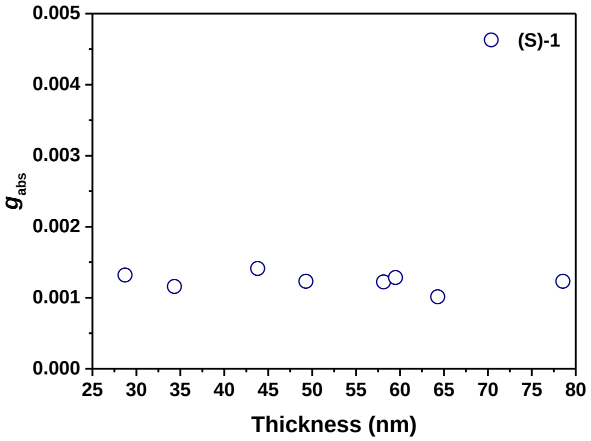

Figure S12. g_{abs} value of **(S)-1** thin films at 385 nm with varying thickness.

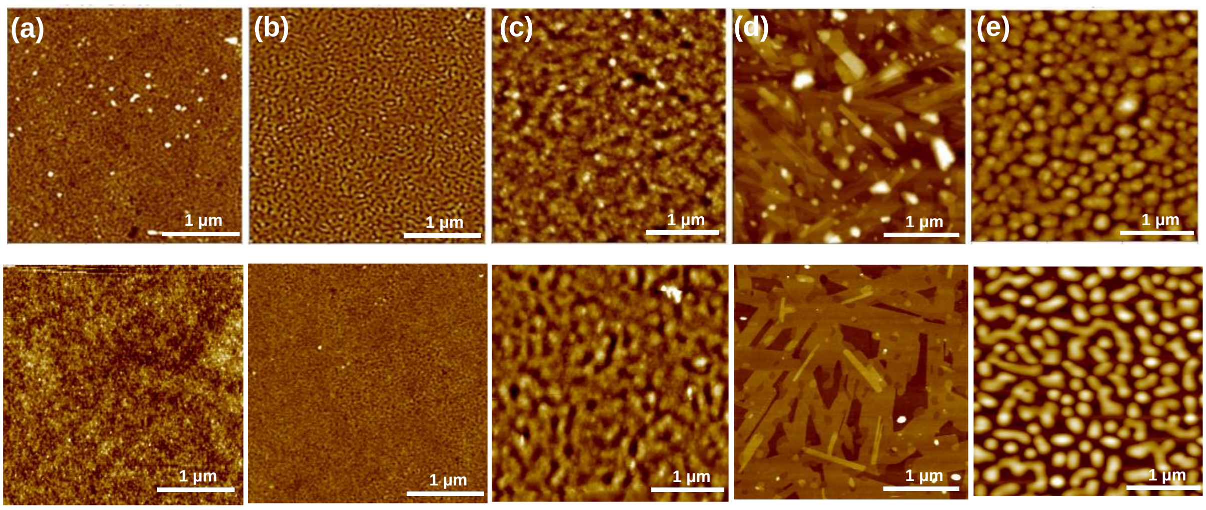

Figure S13. AFM topography images of **(S)-1** (top) and **(R)-1** (bottom) films deposited at a substrate temperature of (a) RT, (b) 75 °C, (c) 125 °C, (d) 160 °C, and (e) 180 °C.

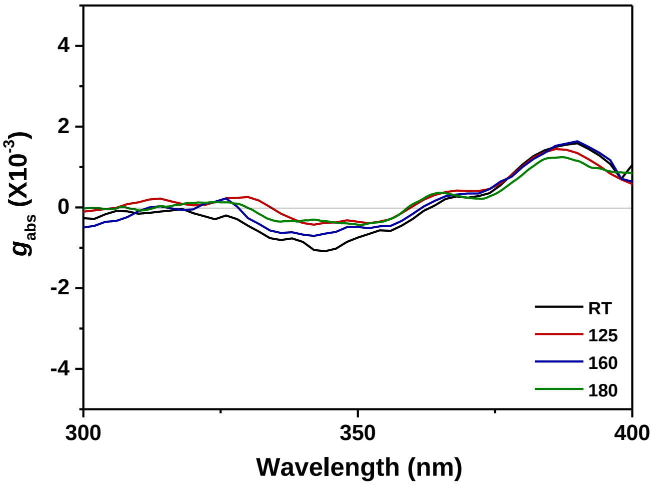

Figure S14. CD spectra of **(S)-1** films deposited at substrate temperature of RT, 125 °C, 160 °C and 180 °C.

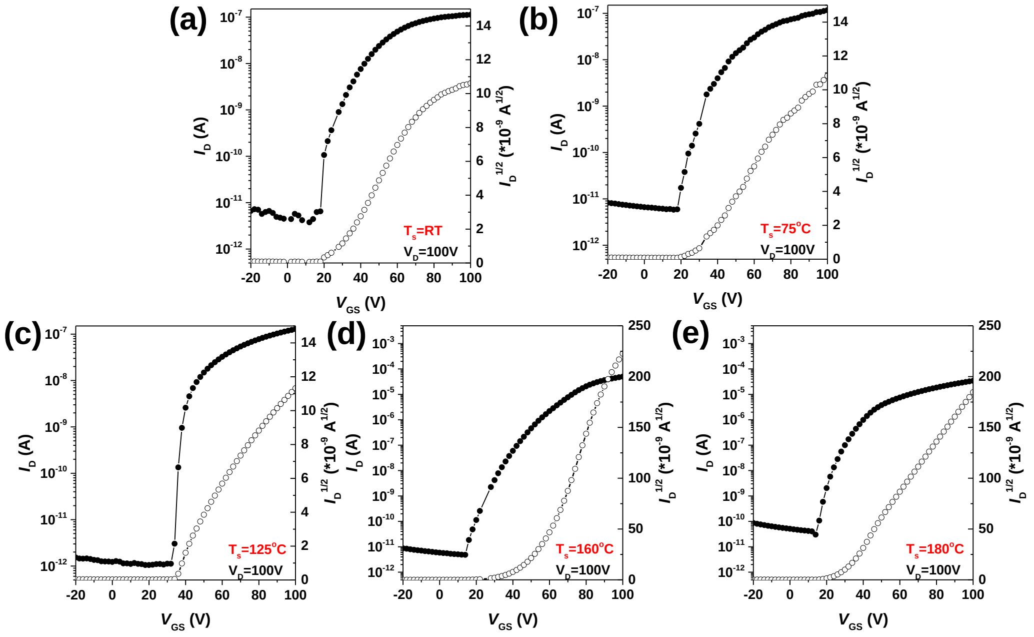

Figure S15. Transfer curves of OFETs based on **(S)-1** films deposited at substrate temperature of (a) RT, (b) 75 °C, (c) 125 °C, (d) 160 °C, and (e) 180 °C (V_{DS}=100 V).

Table S2. OFET performances based on **(S)-1** films deposited at substrate temperature (V_{DS}=100V)

**Substrate temp (°C)**	**μ_{sat} [a] (cm^2 V^{-1} s^{-1})**	**I_{on}/I_{off}**	**V_T [a] (V)**
RT	3.09 (1.06) × 10^{-4}	>10^4	19.1 (1.1)
75	2.86 (0.75) × 10^{-4}	>10^4	18.4 (2.4)
125	4.31 (1.69) × 10^{-4}	>10^4	21.6 (1.6)
160	2.18 (0.56) × 10^{-1}	>10^6	23.3 (0.5)
180	9.65 (7.22) × 10^{-3}	>10^5	32.2 (2.2)

[a] The average and standard deviation values were calculated from more than five OFET devices.

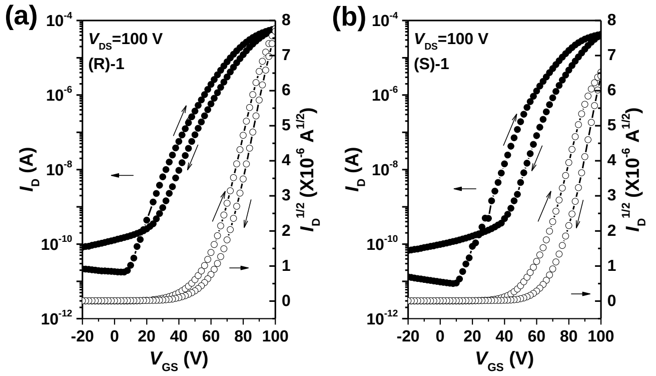

Figure S16. Transfer curves of forward and reverse sweeps of OFETs based on (a) **(R)-1** and (b) **(S)-1** films deposited at substrate temperature of 160 °C.

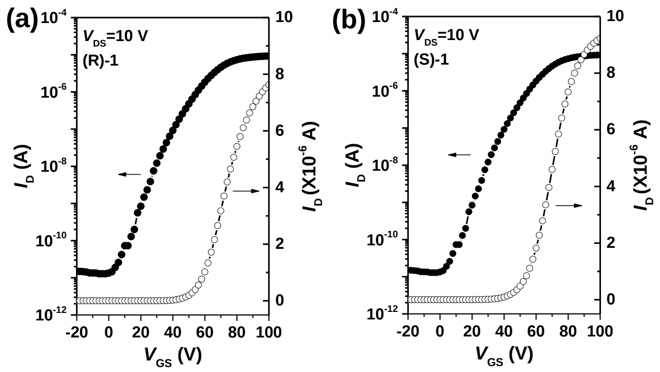

Figure S17. Transfer curves of OFETs based on (a) **(R)-1** and (b) **(S)-1** in the linear regime (V_{DS}=10 V).

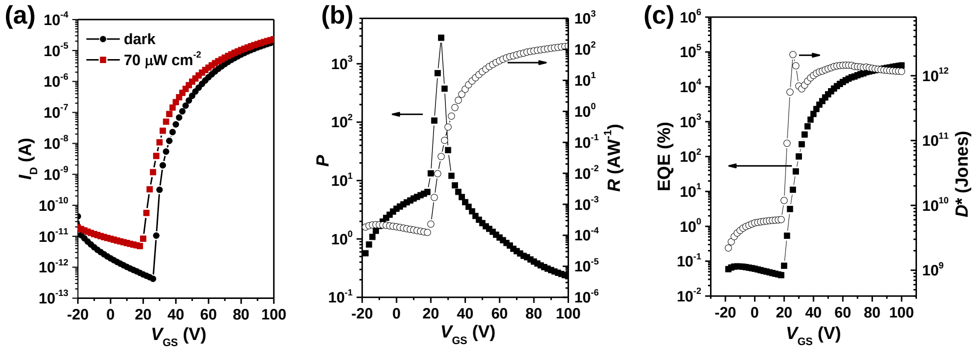

Figure S18. (a) Transfer curves of **(R)-1** films in the dark and under 385 nm UV light irradiations (70 μW cm^{-2}) (b) photocurrent/dark current ratio (*P*), photoresponsivity (*R*) (d) external quantum efficiency (EQE), and detectivity (D^*) of **(R)-1** films under 385nm UV light irradiations (70 μW cm^{-2}).

Table S3. Optoelectronic performances of OPTs based on **(R)-1** and **(S)-1** films.

	μ_{sat}[a] ($cm^2 V^{-1} s^{-1}$)	μ_{lin}[a] ($cm^2 V^{-1} s^{-1}$)	I_{on}/I_{off}	V_T [a] (V)	*P*	*R* ($A W^{-1}$)	EQE (%)	*D** (Jones)
**(R)-1**	3.19 (1.03) x 10^{-1}	1.39 (0.47) x 10^{-1}	>10^6	21.7 (1.6)	2.8 × 10^3	127	4.1 × 10^4	2.2 × 10^{12}
**(S)-1**	2.18 (0.56) x 10^{-1}	1.21 (0.79) x 10^{-1}	>10^6	23.3 (0.5)	1.7 × 10^3	314	8.5 × 10^4	3.9 × 10^{12}

[a] The average and standard deviation values were calculated from more than five OFET devices.

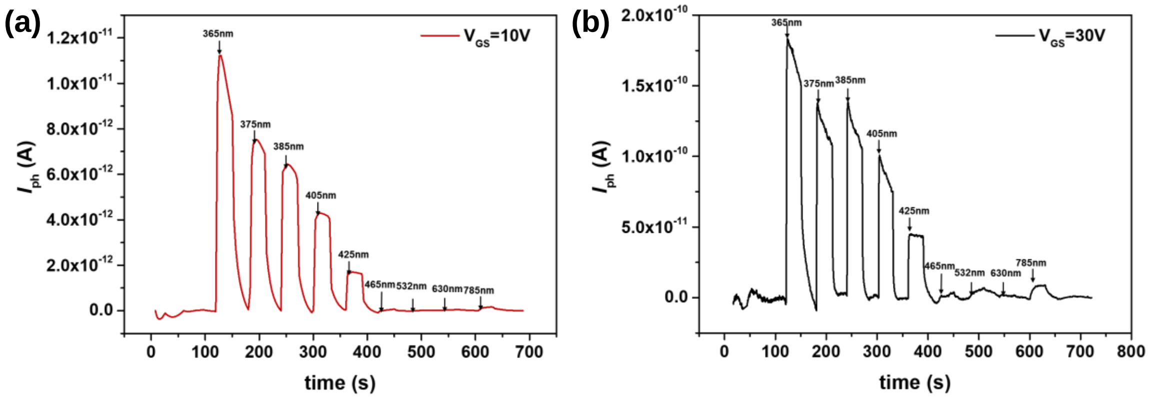

Figure S19. Photocurrent of **(S)-1** films under monochromic light irradiation with different wavelength. (70 μWcm^{-2}).

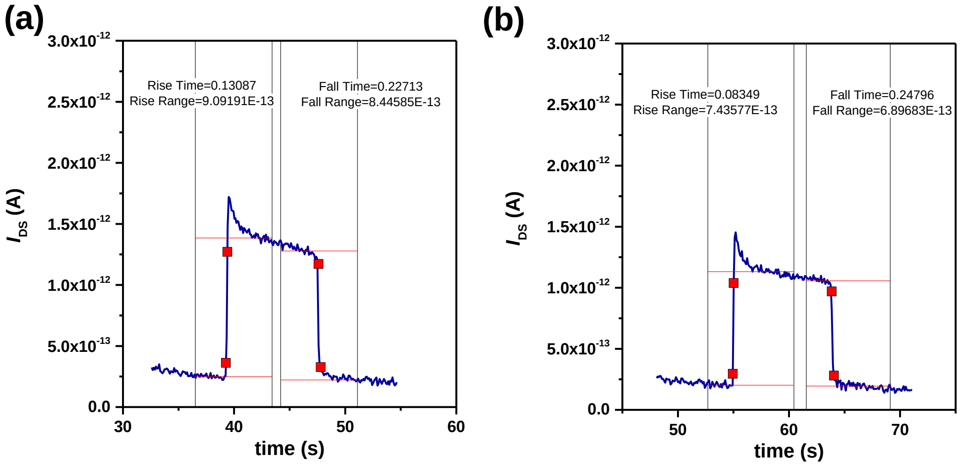

Figure S20. Rise and fall time estimation of (a) **(R)-1** and (b) **(S)-1** thin films under the 385 nm UV light illumination (70 μW cm^{-2}).

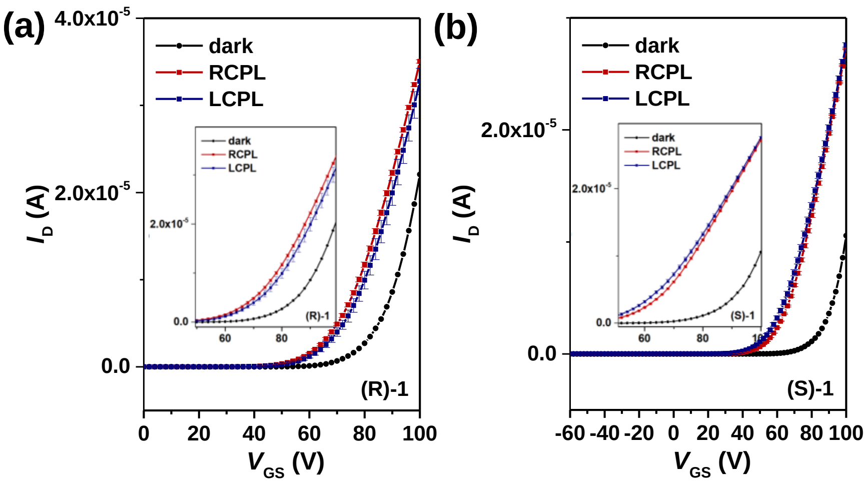

Figure S21. Transfer characteristics of OPTs based on (a) **(R)-1** and (b) **(S)-1** films in dark or under 385 nm CPL illumination (V_{DS}=100 V) and their magnified plot in V_{GS} 50-100 V region (inset).

Table S4. Comparison of this work and other recently published chiral organic small molecule OPTs

Reference	Chiral material	Wavelength	$\|g_R\|$ or $\|g_{ph}\|$	μ ($cm^2 V^{-1} s^{-1}$)	D^* (Jones)
Nat. Photon. **2013**, *7*, 634-638	1-Aza[6]helicene (thin film)	365	-	1.0×10^{-4}	-
Adv. Mater. **2017**, *29*, 16058282	CPDI-Ph (nanowire)	460	-	5.8×10^{-2}	-
ACS Nano **2020**, *14*, 14146-14156	C1CPDI-Ph (supramolecular single crystal)	495	0.12	1.0 (n-doping) 4.9×10^{-3} (intrinsic)	2.1×10^{16}
Adv. Mater. **2021**, *33* , 2004115	(anti,R)16,17-bis[60]PCBM (thin film)	405	1.27	1.44×10^{-5}	-
*Nat. Commun. **2021**, 12,* 1-9.	ortho-π-Extended PDI double-[7]heterohelicene (thin film)	730	0.010	2.1×10^{-3}	2.1×10^{10}
Nat. Commun. **2022**, *13*, 3454	NTPH-P/DPA (heterojunction)	556	0.24	1.1	-
Angew. Chem. Int. Ed. **2023**, 62, 18, e202300972	Dicyanostillbene (supramolecule)	450	0.83	4.4×10^{-2}	1.01×10^{12}
Adv. Mater. **2023**, *35* , 2304627	Fused ring non-fullerene electron acceptor BTP-4F (supramolecular single crystal)	625	1.4	4.4×10^{-1}	3.2×10^{11}
This work	NDI-BN-NDI	385	0.049	2.2×10^{-1}	3.9×10^{12}

References

[S1] C. Jian, T. Tang, *J. Phys. Chem. B* **2014**, *118*, 12772-12780.

[S2] M. J. Abraham, T. Murtola, R. Schulz, S. Páll, J. C. Smith, B. Hess, E. Lindahl, *SoftwareX*, **2015**, 1, 19-25.

[S3] M. Yabe, K. Mori, K. Ueda, M. Takeda, *J. Comput. Chem., Jpn.* **2019**, *5*.

[S4] W. L. Jorgensen, D. S. Maxwell, J. Tirado-Rives, *J. Am. Chem. Soc.* **1996**, *118*, 11225-11236.

[S5] M. D. Hanwell, D. E. Curtis, D. C. Lonie, T. Vandermeersch, E. Zurek, G. R. Hutchison, *J. Cheminf.* **2012**, *4*, 17.

[S6] T. Darden, D. York, L. Pedersen, *J. Chem. Phys.* **1993**, *98*, 10089-10092.

[S7] G. Kresse, J. Hafner, *Phys. Rev. B* **1993**, *47*, 558-561.

[S8] P. E. Blöchl, *Phys. Rev. B* **1994**, *50*, 17953-17979.

[S9] A. H. Larsen, J. J. Mortensen, J. Blomqvist, I. E. Castelli, R. Christensen, M. Dułak, J. Friis, M. N. Groves, B. Hammer, C. Hargus, E. D. Hermes, P. C. Jennings, P. B. Jensen, J. Kermode, J. R. Kitchin, E. L. Kolsbjerg, J. Kubal, K. Kaasbjerg, S. Lysgaard, J. B. Maronsson, T. Maxson, T. Olsen, L. Pastewka, A. Peterson, C. Rostgaard, J. Schiøtz, O. Schütt, M. Strange, K. S. Thygesen, T. Vegge, Lasse Vilhelmsen, Michael Walter, Zhenhua Zeng, Karsten Wedel Jacobsen, *J. Phys. Condens. Matter* **2017**, *29* 273002.

[S10] J. P. Perdew, K. Burke, M. Ernzerhof, *Phys. Rev. Lett.* **1996**, *77*, 3865-3868.

[S11] G. P. Kerker, *Phys. Rev. B* **1981**, *23*, 3082-3084.

[S12] a) A. Morandeira, J. Fortage, T. Edvinsson, L. Le Pleux, E. Blart, G. Boschloo, A. Hagfeldt, L. Hammarström, F. Odobel, *J. Phys. Chem. C* **2008**, *112*, 1721-1728; b) E. Machalska, G. Zajac, M. Baranska, D. Kaczorek, R. Kawęcki, P. F. J. Lipiński, J. E. Rode, J. C. Dobrowolski, *Chem. Sci.* **2021**, *12*, 911-916.
